# Stakeholders’ hopes and concerns about the COVID-19 vaccines in Southeastern Nigeria: a qualitative study

**DOI:** 10.1186/s12889-022-12754-4

**Published:** 2022-02-16

**Authors:** Uchechukwu Madukaku Chukwuocha, Chiagoziem Ogazirilem Emerole, Greg Ndubeze Iwuoha, Ugonma Winnie Dozie, Princewill Ugochukwu Njoku, Chidinma Onyebuchi Akanazu, Precious Okechukwu Eteike, Charlotte Hemingway

**Affiliations:** 1 Department of Public Health, Federal University of Technology, Owerri, Imo State Nigeria; 2 Department of International Public Health, Liverpool School of Tropical Medicine, Liverpool, UK

**Keywords:** COVID – 19, Vaccine, SoutheasternNigeria, Stakeholders, Hopes, Concerns

## Abstract

**Background:**

Equitable access and high uptake of safe and effective vaccines is critical to ending the COVID-19 pandemic. To ensure the success of these vaccines, particularly in many developing and under-developed parts of the world, the concerns of local communities including fears, potency, and levels of acceptance should be addressed. This study assessed community stakeholders’ perceptions in parts of Southeastern Nigeria about COVID-19 vaccine, towards engaging them effectively to ensure the success of the vaccination campaigns.

**Methods:**

A qualitative study was conducted involving fourteen stakeholders from the Southeastern geo-political zone of Nigeria selected using purposive sampling. In-depth semi-structured individual interviews lasting about 30 min were used to collect data. Data analysis was informed by a general inductive approach.

**Results:**

Stakeholders hoped that the development and roll out of the vaccines will bring COVID -19 to an end, will help to maintain good health and allow people get back to normal life. On the other hand, stakeholders expressed their concerns and worries about the “speed” with which the vaccines are being produced, possibility of future adverse effects from vaccination, misinformation, and level of preparedness in the health system to implement the vaccine campaign.

**Conclusions:**

This study identified that more needs to be done to improve perceptions of those who influence health decisions in communities towards COVID-19 vaccines. This includes firstly, the involvement of the community and religious leaders in vaccine promotion. Secondly, it is imperative to develop and disseminate persuasive messaging on vaccine effectiveness and safety targeted at both health professionals, policymakers, and the community which are culturally sensitive and address identified concerns among health workers. Thirdly, the health systems should be strengthened and system-level interventions that directly target one or more of the WHO’s six health system building blocks: service delivery, health workforce, health information systems, access to essential medicines, financing, and leadership/governance.

**Supplementary Information:**

The online version contains supplementary material available at 10.1186/s12889-022-12754-4.

## Background

The COVID-19 pandemic has since 2020 affected over a 100 million people, resulting in more than 2 million global deaths [1]. In Nigeria, about 253,181 cases have been confirmed resulting in about 3,136 deaths [1]. It has also strained health systems, significantly reduced global Gross Domestic Product (GDP), and plunged many countries into economic recession [2–4]. Those identified to be at greater risk of severe illness include the elderly, people with underlying chronic illnesses and immune-suppressed individuals [5–7].

Recommended protective strategies against COVID-19 include the use of face masks, physical distancing and restrictions on social gatherings, and constant hand washing [8]. Although these strategies have proved somewhat effective in curbing the spread of the virus, there has been a resurgence of the disease in several countries, emphasizing the need for more innovative interventions such as vaccines. Effective roll out of vaccines globally, alongside recommended protective strategies, is required to help boost immunity against COVID-19 and mitigate the public health and economic impact of the pandemic.

As of December 2020 [9], about nine vaccines, including the Oxford-AstraZeneca, Pfizer-BioNTech, Moderna, Sputnik V, and Sinopharm had received emergency use listing (EUL) by the World Health Organization (WHO) [10]. The vaccines were at the time recommended for use among those that are eighteen (18) years and older, and particularly the target risk groups including the frontline health workers, those aged sixty (60) years and above as well as those with co-morbidities. These developments, alongside coordination from Gavi and COVAX saw the initiation of a global vaccination campaign against COVID-19 [11], including in several sub-Saharan African (SSA) countries [12]. Nonetheless, challenges, including slow roll out, funding, vaccine safety, and hesitancy among the general population etc., have been identified particularly in SSA countries [13].

In Nigeria, the immediate response to the pandemic was the activation of a nationwide lockdown and restriction in movement, which were eased gradually following attendant economic consequencies on the population. Other measures were soon introduced, including enforcement of handwashing and sanitization as well as marked social distance positions in public spaces. COVID-19 vaccinations in Nigeria began from 5 March 2021 after taking delivery of about 4 million doses of the University of Oxford and AstraZeneca vaccine. As at this time, about 158,042 cases with 1,954 deaths have already been recorded due to the pandemic in the country [13]. It was expected that the vaccines administered at primary health care centers and community headquarters should be mostly made available as soon as possible to the people most at risk ( health workers, the elderly and those with co-morbidities). For the first phase of the COVID-19 vaccine roll-out which mainly targeted frontline health workers and the vulnerable, 98.9% (3,980,600 doses) of the first tranche of Astra Zeneca vaccines was administered on more than 2.5 million persons [14]. However, the uptake of the vaccine has been slow and poor, with 95% of the country’s population yet to receive their first dose as of 17 October, 2021 [14]. A recent study reported issues associated with willingness to receive the COVID-19 vaccine among Nigerians [15]. It is not in the least unexpected. Previous experiences with immunization/vaccination activities in Nigeria indicate widespread vaccine apathy and hesitancy [16, 17].

A typical example is the misconception in Northern Nigeria that the essence of polio vaccines and vaccines in general were part of the western agenda for fertility and population control. This led to poor vaccine uptake in the region and hampered original plans for the elimination of polio [18, 19]. Some of the factors implicated include those related to religion [20, 21], culture [22], health, and safety misconceptions [17, 19, 21, 23]. Furthermore, there is generally very low awareness and uptake of adult vaccinations including those for Hepatitis B, typhoid fever, yellow fever etc. [22]. The COVID-19 vaccines have also been met with certain controversies, particularly due to the "new technology" used in their development which involves the activity of the viral mRNA in the host cell to trigger immunity [24, 25]. As a result of this perceived mechanism of action, some are concerned that the vaccines could manipulate their genetic makeup with adverse consequences [25]. Again the ability of the virus to mutate into different variants is a course for concern for vaccine development and vaccination activities. These concerns could potentially compromise vaccine confidence, hindering the success of vaccinations and the anticipated herd immunity.

The smooth, successful, and sustained rollout of the COVID-19 vaccines hinge on the level of preparedness for its active administration, hence the need for effective and continuous sensitization, mobilization, and vaccine advocacy. These important roles are usually played by local health workers, community leaders and government workers who influence community members on issues bordering health care [26]. Many health interventions fail simply because these stakeholders are not properly engaged during planning and implementation [27]. Meanwhile securing their trust, understanding their hopes and concerns, and putting them into consideration while planning and implementing interventions have been shown to contribute significantly to the success of many health interventions [26, 28].

This qualitative study was therefore aimed at assessing the hopes and concerns of stakeholders in parts of Southeastern Nigeria about the COVID-19 vaccine. Insights from this study were used to establish a set of recommendations to improve understanding and trust among local health workers and community leaders to support implementation of the vaccine programme in Nigeria.

## Methods

### Study design

This was an exploratory qualitative study which used in-depth interviews to gain insights from stakeholders regarding the COVID-19 vaccines in the study area [29]. The study was carried out between January and April 2021 in Owerri, South Eastern Nigeria.

### Study setting

Owerri is the capital of Imo State, South Eastern Nigeria. The town has a diverse demographic population and is densely populated, with a population density of 2,766persons per Km^2^. Owerri, with a distance of 401 km from Abuja, the capital of Nigeria, is a choice destination for vacation and business activities. The inhabitants are mainly public servants, business men, traders and artisans. The communal style of settlement, customs, and traditions found in the area is akin to most of South Eastern Nigeria, thus is a good representation of the wider region. Individuals such as doctors, nurses, pharmacists, drug vendors, religious leaders, community leaders, policy makers and community health worker sare known to influence health decisions including vaccination of the populace and are therefore regarded as stakeholders in the area.

### Recruitment of study participants

A formal enquiry was made at the State Ministry of Health, Owerri, Nigeria to identify the groups of individuals designated as stakeholders who could influence health opinions in communities in the area. Those identified as stakeholders include, the Local Government Area (LGA) department of health resident medical officers, the head of health department in Owerri Municipal Council, the community health extension workers/nurses, traditional rulers, male/female community presidents general and religious leaders in communities of Owerri municipal council, policy makers and the L.G.A Chairman. Based on this, a purposive sampling strategy was employed to achieve variation across the groups identified as stakeholders that could influence health opinions in Owerri municipality, South Eastern Nigeria. This sampling strategy provides an assortment in respondents for gender, age, ethnic and stakeholder type. This strategy is also cost and time effective for this study as restricted movements were still enforced in the State. Being aware that purposive sampling is non–probable and so prone to research bias in sample selection, to reduce this, we had an additional criterion for selection that the stakeholders must have lived in the area for five or more years and understands the population health dynamics in the area. However, four of them could not complete the interviews, leaving us with fourteen stakeholders (Supplement 1). Informed written consent was sought and obtained from them before the interviews were conducted. The criterion for selection was that the stakeholders must have lived in the area for five or more years.

### Data collection

In-depth semi-structured individual interviews lasting about 30 min were used to collect data from the respective stakeholders. The interviews were conducted using a pre-developed tailored topic interview guide developed for the respective stakeholders. Interview continued until saturation was reached when the same comments were being made repeatedly without additional new information being provided. The interview guide was developed through an iterative process involving initial inputs by members of the research team (of diverse public health background), piloting outside Owerri, and a final modification and refinement.

The interviews were conducted by members of the research team who received training on qualitative data collection from an expert from the Ministry of Health, Owerri. All the interviews were conducted in English language and were audio recorded with the consent of the participants. Data was collected between January and February 2021. Data quality was assured by good profiling and checks as well a avoidance of data duplication.

### Data analysis

Using Nvivo version 12, data was analysed by members of the research team following a general inductive approach [30]. Firstly, all audio files were transcribed verbatim by COE using naturalized transcription. Second, UMC and GNI independently read and coded the transcripts and summarised the data. Third, emerging themes were discussed by UMC, GNI, and UWD until agreement was reached on the themes and sub-themes identified. All audio recordings were deleted once transcribed, in keeping with standard ethical practices.

## Results

### Characteristics of study participants

Fourteen stakeholders were enrolled in this study (Supplement 1). They comprised 4 males and 10 females between ages 25 and 55 years. Amongst them were 2 doctors, 3 nurses, 3 drug vendors, 1 policy makers and 3 community health workers, 1 community individual/ leader and 1 religious leader. Their working experiences ranged from 1 to 30 years. A total of 11 participants had tertiary education while the remaining three had secondary education. The highest work experience is 30 years, 11 had at least 5 years experience from health workers, community leader.

### Stakeholder’s hopes and concerns about the COVID-19 vaccine

This study found three broad themes relating to the hopes and concerns of stakeholders regarding the COVID-19 vaccine in South eastern Nigeria. They include:

1. Stakeholder perceptions of current COVID-19 vaccines;
2. Health system preparedness for the COVID-19 vaccination programme;
3. Determinants of COVID-19 vaccine uptake.

The three themes and their subthemes are presented below, together with illustrative quotes.

### Theme 1: Stakeholder perceptions of current COVID-19 vaccines

The unprecedented speed in which the various COVID-19 vaccines have been developed generated both hopes and concerns among the stakeholders. While stakeholdersheld beliefs that vaccines could be effective in conferring immunity against the disease and would lead to positive public health impact there was a clear sense of doubt that effective and safe vaccines could be developed in such a short period of time.

### Perceived benefits of a COVID-19 vaccine

There was a consensus among the stakeholders that the development of vaccines will bring COVID-19 to an end, reduce the death rate as a result of the virus and restore normalcy to daily activities. For some, the vaccine was seen as a means to end face mask mandates enabling the return to ‘*normal life’*:

“I hope the vaccine works and there won’t be any need for hand washing, face masks and we can go back to normal life”(RL)

Furthermore, some of the respondents believed that the development of vaccines will improve the utilization of health care services among individuals at community level; and also improve health servicedelivery by enabling them to treat their patients without ‘*fear’*:

“I expect that it will end the disease, so that people will no longer be scared when they visit drug shops and other health facilities” (DV2)

“I hope that the vaccine ends the disease. I want to treat people without fear” (N1)

In addition, management of new diseases usually poses heavy economic threat at the affected region(s). Thus, reduction of economic and health burden were also identified as one of the expected benefits of aCOVID-19 vaccine.

‘’Vaccine is a welcome development and hopefully it will drive away the disease in our society and reduce the burden and cost that comes with the disease”(PM)

### Concern over the quick emergence of the current COVID 19 vaccines

Various concerns were raised regarding the development of COVID-19 vaccine.Some of the concerns raised by stakeholders centered around the accelerated development of vaccines for COVID-19, consideringthe mean development time for a new anti-infective vaccine is around 10 years and three COVID vaccines were approved for emergency use within 11 months after the SARS-CoV-2 sequence was published.

Some of the stakeholders were aware of the available evidence from WHO and other relevant bodies on the effectiveness and safety of the vaccines. Their level of knowledge could be correlated with the experience that goes with their level or cadre in the health system. However, some of these stakeholders still raised concerns about the quick emergence of the vaccines and the fear for any long term adverse effects including genetic mutation, underpinned by the vaccines use of mRNA technology.

“It is true that WHO and other bodies like CDC have assured us of the vaccines effectiveness and safety. However, when compared to other available vaccines that have stood the test of time, COVID 19 vaccines werethe quickest to emerge” (Dr1).

Other concerns such as uncertainty about the long term adverse effect and/or complications of the vaccine; inadequate information on its appropriateness for vulnerable groups and other conspiracies about its safety were mentioned by the respondents. Of particular concern were pregnant women and children:

“Pregnant women and children should be exempted from vaccination until long term adverse effects (if any) are ascertained. They are the vulnerable groups” (DV3)

### Concern over the neglect of other diseases

There were stakeholders who conveyed their concerns over the neglect of other diseases endemic in the country and factors affecting them as a result of priority attention given to COVID 19. They were bothered that the prevalence of diseases such as malaria, cholera etc. was on the increase because attention has shifted from efforts towards their control to COVID 19. They were of the opinion that COVID 19 was not as serious in the country as it was in other parts of the world, and therefore did not deserve the attention it received over other endemic diseases and hunger. They particularly question why a vaccine has not yet been developed for malaria which they perceived as a more serious disease.

‘'Compared to other countries in the world, we (Nigeria) are not facing worse Covid-19 cases. Other diseases such as malaria still kill more people than Covid-19 does in Nigeria. More people are dying of hunger. While attending to COVID 19,Government should not neglect these other diseases. They should also make the health system functional”(Dr1).

### Theme 2: Health system preparedness for the vaccination program

In this theme, the stakeholders refer methods of vaccine deployment based on previous vaccination programs. These include adequate research and knowledge of the vaccines, human resources, education, and cold chain. Like the previous theme, there were hopes and concerns.

### Hopes for health worker acceptance of the COVID-19 vaccine

Some stakeholders have high hopes that members of the society would be eager to receive the vaccines as many will like to witness normal life activities restored. However, some stakeholders were of the opinion that health workers taking the vaccines first will give others confidence to do the same.

Some stakeholders expressed high levels of self preparedness to receive the vaccine, and encourage other members of the society to do so in order to restore normal life activities in the society.

“I am prepared to receive the vaccine so that those I do attend to in the clinic will have confidence to go for it” (N1)

### Concerns over health workers availability and capacity

Some of the stakeholders who respondedexpressed concerns over perceived inadequacies in the number of available health workers forthe distribution of the vaccines as well asinadequacies in the training of the available health personnel who will administer the vaccine and manage any adverse reactions from vaccination,

“We need to train more hands to be able to distribute this vaccine” (CHW2)

“We need to be adequately informed about this vaccine and how to manage any side effects”(N2)

### Concerns over vaccines supply, storage, and access

It was a concern to some stakeholders that there may not be adequate supply of the vaccines to all Nigerians and that even the available vaccine may not be stored well.

"I don’t know if the vials supplied will be sufficient for our population. " (Dr2)

“I am worried about how cold chain will be maintained to avoid destroying the potency of the vaccine” (Dr2)

### Theme 3: Determinants of COVID-19 vaccine uptake

Stakeholders reported a range of potential enablers and barriers to vaccine uptake, typically drawn from previous experiences in vaccination programs in the region. Some of the stakeholders expressed the need for both verbal and nonverbal persuasion about taking the vaccines. For example, one of the stakeholders shared the perception that people will be convinced to take the vaccine when they see others around them taking it based on their previous vaccination and immunization experiences.

“… you know this is a community. Even if people reject it at first when they see that others who take it are doing well, they will come and take”(CHW1)

Stakeholders were of the opinion that government officials as the leaders should be the first to take the vaccine. This is because they are influential in the communities and thus will motivate others who may be hesitant initially to take the vaccine.

“The government officials should take it first. This will encourage others to do so”(N2)

### Perception on vaccine mandate

In seeking for opinion on whether the COVID 19 vaccines should be made mandatory, a majority of the participants were of the opinion that the vaccines should not be forced or made compulsory, as is seen in other countries but should come as a recommendation.

“We should not be forced to take the vaccine”(Dr1)

“COVID 19 vaccination should come as a recommendation”(PM)

“I don’t think that people should be forced to take the vaccines”(RL)

“Vaccination should not be forced on people”(DV1)

“Since they did not force other vaccines on people, this one should not be different”(C1)

On the contrary, few of the participants suggested that the vaccines should be made compulsory because of the emergency situation posed by COVID-19. They believe that making the vaccines compulsory will help reduce the number of people at risk of getting infected.

“Refusing to take the vaccine will put people at risk. Vaccination should be made compulsory for everybody”(N2)

### Concerns over information sources, information dissemination and conspiracy theory

Stakeholders were also disturbed about the information fatigue and overload from various sources and their subsequent inability to identify the correct and relevant information. They desired to receive COVID-19 related information from credible sources such as the World Health Organization (WHO), the Federal Ministry of Health (FMOH), and the Nigerian Centre for Disease Control (NCDC) as it directly or indirectly influenced their decision to take the vaccine.

“I will like to receive information about COVID via emails from credible sources”(Dr2).

“Information should come from the right channels. From the federal ministry, then to state, then to the local government and to us” (CHW 3).

“The information about the vaccine is not reassuring. We don’t know which one is true or false”(RL)

“ we heard in the media that the vaccine causes blood clots and killing people, and this creates a wrong perception of the vaccine” (DV1)

Another area of concern is the existence of several conspiracy theories which may negatively affect vaccine uptake because the public may likely be susceptible to conspiracy theories underpinned by beliefs in Western tyranny. This particular concern is also subtly raised whenever there is a new vaccination programme particularly for adults.

“The conspiracy theory that Africans or blacks are considered inferior to the whites and treated likewise may make the public believe that the vaccines in the Western world could bedifferent from the one sent to African countries. This will affect the vaccines uptake. (Dr1)”

### Concerns over accessibility and acquisition of vaccines

Nigeria is one of the countries in the world yet to achieve universal health coverage. Most health care expenses are borne out of pocket and road access to health facilities is poor in parts of the country, and this was raised as an issue among the stakeholders with regards to accessibility and possible cost of acquiring the vaccines.

“Access roads to health centers are bad. Most health centers are far and makes it hard to access”(CHW2)

“Healthcare is expensive to get in this country”(CHW1)

“Cost of healthcare in this part is expensive. The patients expect subsidized pay by the government when they visit the health facility. The community people don’t have money to pay for health care, but we try to encourage them to pay for the N100 for deliverables such as cotton wool, etc.” (CHW3).

### Concern over poor working conditions and welfare of health workers

Lack of motivation amongst stakeholders was reported by the respondents. These include none payment of salaries and allowances, none provision of protective equipment, in-conducive working conditions, to mention a few.

“We are being owed salary for months and we need these things to motivate us to work’’ (N1)

“……… what did the government give us? Nothing. Just one bottle of hand sanitizers” (N3)

## Discussion

The Study employed qualitative techniques to uncover stakeholders’ hopes and concerns about the COVID-19 vaccine in parts of South Eastern Nigeria. Considering the disruptions in normal daily life activities caused by the pandemic, the stakeholders hoped that the vaccine could help them return to normalcy. Also, the spread of the virus across nations since its outbreak has been unprecedented thus causing panic among individuals both in the health system and beyond. COVID-19 vaccine administration is therefore expected to create herd immunity. However, herd immunity can only be achieved when a good number of people have been vaccinated or infected and recovered. This will obviously be achieved faster with vaccination. In a similar way, most of the stakeholders who responded expressed hope in seeing the vaccine reduce the mortality rate of the disease as well as reduce its spread across states.

Additionally, SARS-CoV-2 being a novel strain of the corona virus has posed a challenge in its management and /or treatment since its outbreak in 2019. Consequently, utilization of health care services has witnessed a significant drop since the onset of the pandemic. Vaccine development and further research on the virus will, however,improve strategies in managing patients with the virus and also improve the use of available health care services. Majority of the stakeholders who responded expressed hope in seeing people using health care services without the fear of being infected by the virus. Also, they expected the vaccine to make healthworkers less vulnerable to the virus thus improving their ability to efficiently execute quality health care services to patients.

However, while stakeholders expressed their hopes to the vaccine development, several concerns were also raised regarding vaccine effectiveness, accelerated development, possible adverse effects, and willingness to get vaccinated. According to the International Federation of Pharmaceutical Manufacturers & Associations [31], vaccine production usually takes 10–15 years and also costsabout US$31–68 million. But due to the heavy global disruption caused by the virus and persistent increase in the number of infection, there was a call to accelerate the production and rollout of vaccines against COVID-19 [7, 32, 33]. Despite this obvious need, there are apprehensions in the population surrounding the efficacy, accessibility and distribution of the vaccine. In a similar way, the prevailing lack of information and misinformation about COVID-19 could have an overbearing influence on what people believe or not regarding the vaccines [34]. Some of the stakeholders revealed their astonishment at the cogency and the magnitude of attention devoted to COVID-19 by the government, insisting that such concerted efforts have not been geared towards solving other prevalent problems in the country. This makes it difficult for them to regard the efforts being made by government towards COVID 19 vaccination as credible. These concerns are apt in the sense that the government has been viewed as being less concerned in tackling the associating problems of hunger, poverty, insecurity, and other social issues affecting the country.

This study also reveals meaningful insights on stakeholders’ inputs for preparedness for COVID-19 vaccine and the results obtained can be integrated with other outcomes for successful vaccination. Ways recommended to build public trust and prime the vaccines include human resource development to improve self-efficacy of those going to administer the vaccines as well as adequate sensitization on the mechanisms and potential side effects of the vaccines, taking good care to avoid the adulteration and loss of integrity. Others include making the vaccine affordable and accessible, and putting in place efficient structures for the preservation of the vaccine to avoid wastage are ways recommended to build public trust and prime the vaccine rollout for success. Oku et al. [26] identified similar reasons in their study on factors affecting the implementation of childhood vaccination communication strategies in Nigeria.

Furthermore, some of the stakeholders perceived COVID-19 as being given an exaggerated priority. This can be a possible avenue to misappropriate funds. This finding is quite similar to responses in earlier studies [35, 36]. Moreover, tackling the social determinants of health is albeit important towards improving responses from the population concerning COVID-19 testing and preventive techniques [37], and could as well be a requisite for vaccine success. Another major concern raised is the mistrust and confusion surrounding the long term effects of the vaccines. A prevailing misconception is that the vaccines could be a means of population control. Such conspiracy theories have also trailed the origins of COVID-19. Reasons for the mistrust could be as a result of little efforts to dispel misconceptions and instill the necessary confidence particularly among stakeholders. Similar to the findings in another study [36], some of the stakeholders in the index study are concerned that the vaccines shipped to Africa could be for human experiments. It has been reported that COVID-19 conspiracy theories negatively influence the adoption of protective measures against the pandemic, and the willingness to get vaccinated against the disease [38].

## Conclusion

In conclusion, while stakeholders have high expectations that the development and rollout of COVID-19 vaccines could reduce the disease and restore normal life activities, some were opinion reserved and could not openly express their preparedness in getting vaccinated for COVID-19. This study identified that more needs to be done to improve health worker and community perceptions towards COVID-19 vaccines to ensure success in the vaccination campaign. This include firstly, the involvement of community and religious leaders in vaccine promotion [19, 39]. Secondly, it is imperative to develop and disseminate persuasive messaging on vaccine effectiveness and safety targeted at both health professionals, policy makers and the community which are culturally sensitive, and address identified concerns among health workers. Thirdly, the health systems should be strengthened and system-level interventions that directly target one or more of the WHO’s six health system building blocks: service delivery, health workforce, health information systems, access to essential medicines, financing, and leadership/governance, developed. Disease-specific interventions that have important system-wide effects to support vaccine roll out should be put in place. Fourthly, relevant stakeholders are not effectively engaged in vaccination activities in Nigeria, as is the case with the COVID-19 vaccines in the study area. It is therefore important that relevant stakeholders who are able to influence public opinions and behaviours regarding vaccinations, are engaged right from the planning stages to implementation and follow up stages to ensure effective coverage and sustianable vaccination programmes.

### Limitations

The reported study was not without limitation. Purposively sampled stakeholders predominantly operated at the lower levels of the health system (community/dispensary), as such the reported findings should not be considered as representative of the entire health system in Nigeria. Perceptions from potentially important stakeholders such as national level policy makers, representatives from the private sector and COVAX were not captured. Having said that, the focus on stakeholders from lower levels of the health system was important as they have direct influence on community perceptions and behaviour towards vaccines. To increase the generalizability of the results, we recommend that this study be conducted among additional stakeholders and in other parts of the country.

Trustworthiness of data collected was ensured throughout the study duration by a study code assigned to each respondent at the time of analysis. All data was handled in the strictest confidence. In addition, interviewers did not participate in the discussions so as not to introduce bias to responses.

## Supplementary Information

**Additional file 1.**

## Data Availability

The datasets used and/or analysed during the current study are available from the corresponding author on reasonable request.
