# Supplementary material for: Stakeholders’ hopes and concerns about the COVID-19 vaccines in Southeastern Nigeria: a qualitative study
Source: BMC Public Health. 2022 Feb 16;22:330. doi: 10.1186/s12889-022-12754-4 (PMC8848682; doi:10.1186/s12889-022-12754-4)
Supplement: Supplementary file 1 — Additional file 1. [file 12889_2022_12754_MOESM1_ESM.docx]

**Supplement 1: Characteristics of the Stakeholders (n = 14)**

| **Code** | **Age** | **Gender** | **Designation** | **Education** | **Years of Work Experience** |
| --- | --- | --- | --- | --- | --- |
| Dr1 | 45 | Male | Chief Consultant Medicine | Tertiary | 22 years |
| Dr2 | 55 years | Female | Chief Consultant Obstetrics and Gynaecology | Tertiary | 30 years |
| CHW1 | 45 years | Female | Community Health Worker | Tertiary | 19 years |
| N1 | 52 | Male | Nurse | Tertiary | 8 years |
| N2 | 52 | Female | Nurse | Tertiary | 5 years |
| N3 | 25 | Female | Nurse | Tertiary | Less than 1 year |
| CHW2 | 42 | Female | Community Health Worker | Tertiary | 1 year |
| CHW3 | 43 | Female | Community Health Worker | Tertiary | 7 years |
| DV1 | 25 | Female | Drug vendor | Secondary | 9 years |
| DV2 | 26 | Female | Drug vendor | Secondary | 2 years |
| DV3 | 44 | female | Drug vendor | Tertiary | 5 years |
| PM | 53 | Female | Supervisor of Health/Policy Maker | Tertiary | 10 years |
| RL | 40 | Male | Religious Leader | Tertiary | 20 years |
| CI | 30 | Male | Community Individual | Secondary | 5 years |

**Supplement 2. Summary of Themes and Sub Themes**

| **Main theme** | **Hopes** | **Concerns** |
| --- | --- | --- |
| Stakeholder perceptions of current COVID-19 vaccines | - Perceived benefits of a COVID-19 vaccine | - Concern over the quick emergence of the current COVID 19 vaccines - Concern over the neglect of other diseases |
| Health System Preparedness for the Vaccination Program | - Hopes for health worker acceptance of the COVID-19 vaccine | - Concerns over health workers availability and capacity - Concerns over vaccines supply, storage, and access |
| Determinants of COVID-19 Vaccine Uptake | - Perception on vaccine mandate | - Concerns over information sources and dissemination and conspiracy theory - Concerns over accessibility and acquisition of vaccines - Concern over poor working conditions and welfare of health workers |
